# Offspring sex ratio and gonadal irradiation in the British Childhood Cancer Survivor Study

**DOI:** 10.1038/sj.bjc.6603736

**Published:** 2007-04-10

**Authors:** R C Reulen, M P Zeegers, E R Lancashire, D L Winter, M M Hawkins

**Affiliations:** 1 Centre for Childhood Cancer Survivor Studies, Department of Public Health and Epidemiology, University of Birmingham, Edgbaston, Birmingham B15 2TT, UK; 2 Unit of Genetic Epidemiology, Department of Public Health and Epidemiology, University of Birmingham, Edgbaston, Birmingham B15 2TT, UK; 3 Department of General Practice, Comprehensive Cancer Institute Limburg, Catholic University of Leuven, Leuven, Belgium

**Keywords:** radiation, sex ratio, childhood cancer survivors, epidemiology

## Abstract

We investigated offspring sex ratio among 6232 offspring born to 3218 survivors of childhood cancer in relation to therapeutic irradiation, and pooled our data with those from two other large-scale studies giving a total of 9685 offspring. Exposure to high-dose gonadal irradiation was not associated with a significant alteration in offspring sex ratio compared to low doses (men: *P*=0.58, women: *P*=0.66). There was also no evidence that the ratio varied with time since cancer diagnosis when comparing survivors treated with radiotherapy *vs* those without (men: *P*=0.51; women: *P*=0.46). This, the largest study to date, finds no evidence that exposure to radiation affects the offspring sex ratio among survivors of childhood cancer.

Several studies (Schull and Neel, 1958; Schull *et al*, 1966; Hawkins, 1991; Dickinson *et al*, 1996; Byrne *et al*, 1998; Maconochie *et al*, 2001; Green *et al*, 2003; Winther *et al*, 2003) have investigated whether parental preconceptional exposure to radiation alters the sex ratio (boy/girl ratio) of offspring, but most (Schull *et al*, 1966; Hawkins, 1991; Byrne *et al*, 1998; Maconochie *et al*, 2001; Green *et al*, 2003; Winther *et al*, 2003) have failed to find supporting evidence. This may be because offspring sex ratio is not a sensitive indicator of radiation-induced genetic damage (Neel *et al*, 1990). It is also possible, however, that the studies lacked adequate statistical power. Survivors of childhood cancer provide a unique opportunity to investigate the effect of radiation on sex ratio because a large proportion of survivors have been exposed to high doses of therapeutic radiation (Boice *et al*, 2003). We have investigated the subject within a large-scale cohort study of adult survivors of childhood cancer, providing us with more offspring born to childhood cancer survivors compared to the largest of the previous studies, which investigated the effect of gonadal dose (Byrne *et al*, 1998), and pooled our data with those from previous large-scale studies, in order to maximise statistical power.

## MATERIALS AND METHODS

### Study design

We used data from the *British Childhood Cancer Survivor Study* (BCCSS), which is a population-based cohort study of survivors of childhood cancer who were 16 years or older, had been diagnosed with childhood cancer between 1940 and 1991, in Britain, and who had survived for at least 5 years. As part of the BCCSS, a questionnaire that included details of adverse health outcomes and the sex of any offspring was sent to 14 540 survivors in the cohort. In total, 10 477 returned the questionnaire, yielding a response rate of 72%. Twenty-seven per cent reported having produced at least one child, with a total of 5433 offspring born to 2793 survivors. As part of an earlier investigation (Hawkins, 1991), General Practitioners of 2286 survivors were sent a questionnaire that also enquired about the sex of any survivor offspring. Although most survivors included in this latter study were also included in the more recent comprehensive investigation, 425 survivors did not complete the more recent questionnaire, but their GP at the time had provided us with information on their offspring. This allowed us to add 799 offspring to the existing 5433 offspring available for this investigation, giving a total number of 6232 offspring born to 3218 survivors.

Data relating to cancer type and site, radiotherapy treatment (yes/no), chemotherapy treatment (yes/no), and demographics were obtained from the *National Registry of Childhood Tumours*. The information on tumour site, together with information on whether the survivor had been treated with radiotherapy, allowed us to determine whether the gonads of the survivor had been exposed to relatively high or low doses of radiation. Treatment with radiotherapy was considered to result in a relatively high gonadal dose if the site of the irradiated tumour had been between the knee and the diaphragm (hereafter *high gonadal dose*); treatment was presumed to result in a relatively low gonadal dose if the tumour had not been irradiated or the irradiation had occurred below the knee or above the diaphragm (hereafter *low gonadal dose*).

### Statistical analysis

We expected that radiation induced sex-linked lethal mutations would manifest as an increase in the offspring sex ratio in male survivors and as a decrease in female survivors (Schull and Neel, 1958). First, we calculated the offspring sex ratio for all childhood cancer survivors and compared it with the offspring sex ratio observed in the general population of England and Wales (Office of National Statistics, 2002). We then compared the offspring sex ratio of survivors treated with chemotherapy *only*, or radiotherapy *only*, with that of survivors treated without chemotherapy or radiotherapy. However, because not all radiotherapy is likely to have resulted in a high gonadal dose, we calculated the offspring sex ratio of survivors who had been treated with a high gonadal dose and the offspring sex ratio of survivors who had been treated with a low gonadal dose. Subsequently, we estimated the odds ratio (OR), which expressed the offspring sex ratio (i.e. the odds of offspring being of male sex) of those treated with a high gonadal dose relative to that of those treated with a low gonadal dose by means of unconditional logistic regression, while adjusting for the potential confounders of *treatment with chemotherapy*, *parental age* (in 5-year groups), and *birth order*. However, as this adjustment left the estimates unchanged, we report only crude ORs.

As radiation-induced germ cell mutations may be repaired or be followed by cell death, an observed effect may vary with time. We therefore examined whether the association, if any, between treatment with radiotherapy and offspring sex ratio varied by the time interval between diagnosis and birth of offspring. Specifically, we calculated ORs comparing survivors exposed to radiotherapy *only* with survivors not exposed to radiotherapy or chemotherapy according to different levels of the time interval between diagnosis and birth of offspring (<10/10–14/15–19/⩾20 years), and tested whether these ORs varied significantly from each other. In addition, we performed a test for trend to examine whether the log odds of offspring being of male sex increased or decreased linearly over the different categories of this time interval.

In addition, the findings from this study were pooled with those from studies by Winther and Byrne (Byrne *et al*, 1998; Meistrich and Byrne, 2002; Winther *et al*, 2003). Byrne *et al* (1998) defined potential mutagenic irradiation as treatment with radiotherapy above the knee and below the diaphragm, and therefore we considered these survivors as being exposed to high-dose gonadal irradiation. Survivors treated with alkalyting agents were also included in this group, because, in their calculation of the sex ratio, Byrne *et al* (1998) did not distinguish between those survivors treated with high gonadal radiation and those treated with alkalyting agents as both treatments were hypothesised to be potentially germ-cell mutagenic. Winther *et al* (2003) used a classification scheme from low to high to categorise survivors by the likelihood of the gonads having been exposed to therapeutic irradiation. To pool our data with this latter study, we regarded the category defined as high as that in which survivors had been exposed to high gonadal doses.

## RESULTS

The sex ratio for offspring of male and female survivors was 1. 02 (95% CI: 0.94, 1.10) and 1.08 (95% CI: 1.01–1.15), respectively, which is indistinguishable from the sex ratio of 1.05 observed in the general population of England and Wales. For male and female survivors, no significant difference (*P*=0.69; *P*=0.72, respectively) was found between the sex ratio of those treated with radiotherapy *only vs* survivors treated without any radiation or chemotherapy (Table 1). Neither was treatment with chemotherapy *only* associated with a significant alteration in sex ratio in males (*P*=0.98) or females (*P*=0.99). Also no statistically significant difference in sex ratio between the offspring of survivors of each sex treated with high-gonadal dose irradiation *vs* those treated with low-gonadal dose irradation (males: *P*=0.58; females: *P*=0.66). For survivors exposed to high gonadal doses, the most common diagnosis was Wilms’ tumour (62.7%), followed by bone tumour (9.7%) and soft-tissue sarcoma (8.3%).

The sex ratio did not vary significantly over time intervals between diagnosis and birth of offspring when comparing survivors treated with radiotherapy *only* with those treated without (males: *p*_heterogeneity_=0.51, females: *p*_heterogeneity_=0.46). There was also no significant linear relationship between offspring sex ratio and time interval between diagnosis and birth of offspring (males: *p*_trend_=0.46, females: *p*_trend_=0.74). For the three studies combined, sex ratios did not differ significantly for survivors treated with high dose gonadal irradiation *vs* those treated with low gonadal doses ((males: *P*=0.94), females: *P*=0.31) Table 2.

## DISCUSSION

This study, which was based upon a greater number of cancer survivor offspring than available from any previous study, found no evidence for an altered sex ratio among the offspring of survivors of childhood cancer previously treated with high-dose gonadal irradiation. These findings are consistent with an earlier report from Denmark by Winther *et al* (2003). Although non-significant, Byrne *et al* (1998) and Meistrich and Byrne (2002) previously reported a sex ratio of 0.84 among female survivors who had been treated with potentially mutagenic therapy *vs* a sex ratio of 1.03 among those who had been treated with presumed less or non-mutagenic therapy. However, Byrne *et al* (1998) also included survivors who had been treated with potentially mutagenic-alkylating agents. We were unable to investigate the separate effects of these agents, which may explain the small difference with our findings. Consistent with the findings of Winther *et al* (2003), we did not find evidence that the offspring sex ratio changes with time since cancer diagnosis.

A limitation of this study might be that information on the offspring sex ratio was based on questionnaire data and, inherent to such an approach, the fact that not all survivors responded. However, the high proportion of eligible survivors returning a questionnaire, together with the fact that respondents were similar to nonrespondents with regard to treatment factors, gives grounds for regarding response bias as not important.

Another limitation might be related to other known or unknown factors influencing the offspring sex ratio such as, for example, time of conception on different days of the menstrual cycle (Harlap, 1979), though it is unlikely that such factors would confound the association between exposure to gonadal irradiation and offspring sex ratio.

Apart from the atomic bomb survivor study (Schull *et al*, 1966), most studies conducted to date have either included relatively few offspring of parents who were exposed to potentially germ-cell mutagenic irradiation, or the offspring were produced by parents who were exposed to relatively low gonadal doses of irradiation compared with childhood cancer survivors. The strength of this study is the large number of offspring born to survivors (*n*=6232), more than 2.5-fold that included in the largest previous study (*n*=2198) (Byrne *et al*, 1998; Meistrich and Byrne, 2002) that investigated the effect of gonadal dose. Even with these numbers, insufficient statistical power may have led to some small alterations in the sex ratio going undetected. However, as observed by Neel *et al* (1990), it is probable that sex ratio is not a sensitive indicator of radiation-induced genetic damage in humans. The discovery of the Lyonisation phenomenon, which established that, in women, only one X-chromosome in each somatic cell is actually active, may be relevant. By pooling our data with those from previous studies, we have maximised the statistical power available to address the variation in sex ratio, and we have reduced the plausible size of any hypothesised effect.

## Figures and Tables

**Table 1:** Offspring sex ratio by sex of parent according to different types of treatment

	**Male survivors**	**Female survivors**
All survivors	1.02 (1221/1198)^a^	1.08 (1725/1858)^a^
Treated with RT only	0.95 (436/457)	1.09 (485/445)
Treated without RT or CT	1.00 (278/279)	1.13 (496/440)
OR (95% CI)^b^	0.96 (0.77, 1.18)	0.97 (0.81, 1.16)
Treated with CT only	1.00 (92/92)	1.13 (152/135)
Treated without CT or RT	1.00 (278/279)	1.13 (496/440)
OR (95% CI)^c^	1.00 (0.72, 1.40)	1.00 (0.77, 1.30)
High-dose gonadal RT	0.96 (140/146)	1.03 (187/181)
Low-dose gonadal RT	1.03 (1058/1029)	1.08 (1628/1501)
OR (95% CI)^d^	0.93 (0.73, 1.19)	0.95 (0.77, 1.18)

CI=confidence interval; CT=chemotherapy; OR=odds ratio; RT=radiotherapy.

Number of boys *vs* girls given in paranthesis.

a Total number of offspring was 6232, but for 230 offspring sex was unknown.

b OR expressing the sex ratio for those treated with RT *only* *vs* those treated without RT or CT.

c OR expressing the sex ratio for those treated with CT *only* *vs* those treated without RT or CT.

d OR expressing the sex ratio for those treated with high-dose gonadal RT *vs* those with low doses.

**Table 2:** Sex ratio of survivors treated with potentially high-dose gonadal irradiation *vs* those treated with presumed low-dose gonadal irradiation, based on pooling of data from the current study, that of Winther *et al* (2003) and that of Byrne *et al* (1998)

	**Boys/girls ratio among survivors treated with**		
**Sex survivor**	**High gonadal dose**	**Low gonadal dose**	**OR^a^ (95% CI)**
Males	1.01 (272/269)^b^	1.02 (1773/1742)^b^	0.99 (0.83, 1.19)
Females	0.97 (308/318)^b^	1.06 (2570/2433)^b^	0.92 (0.78, 1.08)

CI=confidence interval; OR=odds ratio.

a ORs expressing the sex ratio of those treated with high-gonadal dose radiation *vs* those treated with low doses.

b No. of boys/girls.
